# Supplementary material for: Identification of photoperiod-induced specific miRNAs in the adrenal glands of Sunite sheep (Ovis aries)
Source: Front Vet Sci. 2022 Jul 22;9:888207. doi: 10.3389/fvets.2022.888207 (PMC9354845; doi:10.3389/fvets.2022.888207)
Supplement: Supplementary file 1 [file Data_Sheet_1.ZIP › Supplementary file/miRDeep2 file.zip!/miRDeep2 file/Know_Structure/SP42A3/oar-let-7g.pdf]

miRBase precursor : oar-let-7g
Total read count : 812442
oar-let-7g read count : 812423
remaining reads : 19

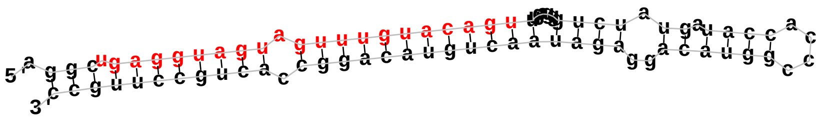

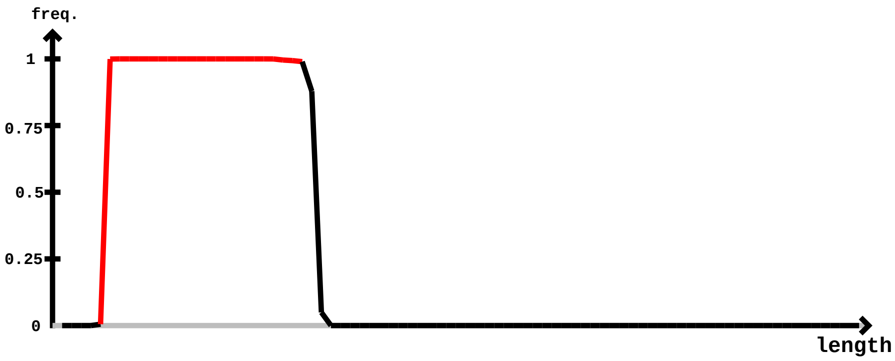


```
      oar-let-7g
5'- aggcugagguaguaguuuguacaguuugagggucuaugauaccacccgguacaggagauaacuguacaggccacugccuugcc  -3'    exp
    .(((.((((((((.(((((((((((.....((((.((.((((....))))))..))))))))))))))).)))))))))))   reads     mm        sample
    ...cugagguaguaguuugua..............................................................   1         0         seq
    ...cugagguaguaguuuguac.............................................................   30        0         seq
    ...cugagguaguaguuuguaca............................................................   15        0         seq
    ...cugagguaguaguuuguacag...........................................................   21        0         seq
    ...cugagguaguaguuuguacagu..........................................................   666       0         seq
    ...cugagguaguaguuuguacaguu.........................................................   3265      0         seq
    ...cugagguaguaguuuguacaguuu........................................................   204       0         seq
    ....ugagguaguaguuuAua..............................................................   1         1         seq
    ....ugagguaguaguuugGa..............................................................   1         1         seq
    ....ugagguaguaguuugua..............................................................   114       0         seq
    ....ugagguaguaguuuguac.............................................................   3282      0         seq
    ....ugagguagCaguuuguac.............................................................   8         1         seq
    ....ugagguaguaguuuguaca............................................................   1784      0         seq
    ....ugagguaguaguuuguacag...........................................................   3160      0         seq
    ....ugagguaguaguuuguacagu..........................................................   89215     0         seq
    ....ugagguaguaguuuguacaguu.........................................................   670286    0         seq
    ....ugagguaguaguuuguacaguuu........................................................   39889     0         seq
    ....ugagguaguaguuuguacaguuug.......................................................   24        0         seq
    ....ugagguaguaguuuguacaguuuga......................................................   5         0         seq
    .....gagguaguaguuuguac.............................................................   1         0         seq
    .....gagguaguaguuuguacag...........................................................   1         0         seq
    .....gagguaguaguuuguacagu..........................................................   51        0         seq
    .....gagguaguaguuuguacaguu.........................................................   367       0         seq
    .....gagguaguaguuuguacaguuu........................................................   29        0         seq
    ......agguaguaguuuguacagu..........................................................   1         0         seq
    ......agguaguaguuuguacaguu.........................................................   1         0         seq
    ........guaguaguuuguacaguu.........................................................   1         0         seq
    .........uaguaguuuguacaguuugagg....................................................   1         0         seq
    .......................................uaccacccgguacaggagauaa......................   1         0         seq
    ...........................................................aacuguacaggccacugccuugc.   4         0         seq
    ............................................................acuguacaggccacugccuugc.   2         0         seq
    .............................................................cuguacaggccacugccuug..   1         0         seq
    .............................................................cuguacaggccacugccuugc.   5         0         seq
    .............................................................cuguacaggccacugccuugcc   5         0         seq
```
